# Modeling the COVID-19 Epidemic With Multi-Population and Control Strategies in the United States

**DOI:** 10.3389/fpubh.2021.751940

**Published:** 2022-01-03

**Authors:** Deshun Sun, Xiaojun Long, Jingxiang Liu

**Affiliations:** ^1^Shenzhen Key Laboratory of Tissue Engineering, Shenzhen Laboratory of Digital Orthopedic Engineering, Guangdong Provincial Research Center for Artificial Intelligence and Digital Orthopedic Technology, Shenzhen Second People's Hospital (The First Hospital Affiliated to Shenzhen University, Health Science Center), Shenzhen, China; ^2^School of Marine Electrical Engineering, Dalian Maritime University, Dalian, China

**Keywords:** COVID-19, mathematical model, parameter estimate, sensitive analysis, control strategies

## Abstract

As of January 19, 2021, the cumulative number of people infected with coronavirus disease-2019 (COVID-19) in the United States has reached 24,433,486, and the number is still rising. The outbreak of the COVID-19 epidemic has not only affected the development of the global economy but also seriously threatened the lives and health of human beings around the world. According to the transmission characteristics of COVID-19 in the population, this study established a theoretical differential equation mathematical model, estimated model parameters through epidemiological data, obtained accurate mathematical models, and adopted global sensitivity analysis methods to screen sensitive parameters that significantly affect the development of the epidemic. Based on the established precise mathematical model, we calculate the basic reproductive number of the epidemic, evaluate the transmission capacity of the COVID-19 epidemic, and predict the development trend of the epidemic. By analyzing the sensitivity of parameters and finding sensitive parameters, we can provide effective control strategies for epidemic prevention and control. After appropriate modifications, the model can also be used for mathematical modeling of epidemics in other countries or other infectious diseases.

## Introduction

Coronavirus disease-2019 (COVID-19) is a contagious disease caused by severe acute respiratory syndrome coronavirus 2 (SARS-CoV-2). Anyone can have mild to severe symptoms. Older adults and people who have severe underlying medical conditions like heart or lung disease or diabetes seem to be at higher risk of developing more serious complications from COVID-19 illness. People with COVID-19 have had a wide range of symptoms reported ranging from mild symptoms to severe illness. Symptoms may appear 2–14 days after exposure to the virus. People with these symptoms may have COVID-19: fever or chills, cough, shortness of breath or difficulty breathing, and so on (1).

The first case was identified in Wuhan, China, in December 2019. It has since spread worldwide, leading to an ongoing pandemic. As of January 19, 2021, the total number of confirmed cases worldwide has reached 96,158,807, and the death toll has reached 20,570,050. Among them, the cumulative number of people infected with COVID-19 in the United States is as high as 24,433,486, and the number is still rising. In addition, with the emergence of mutant virus strains in the United Kingdom that spreads more easily and quickly than the other variants (2), it may make the epidemic prevention situation more severe. Mathematical modeling is one of the most effective methods for forecasting of infectious disease outbreaks and, thus, yielding valuable insights to suggest how future efforts may be improved. An important method for epidemiological studies of such acute infectious diseases is mathematical modeling (3–6). Therefore, it is necessary to establish a mathematical model to accurately predict the evolution trend of COVID-19 in the United States, and find key factors that can significantly affect the evolution of COVID-19 to provide effective control strategies.

At first, many scholars established mathematical modeling research on COVID-19 in China (7–12). For example, Wu et al. (8) proposed a four-dimensional ordinary differential equations to research on the epidemic in Wuhan at the beginning, and the estimated basic reproductive number was 2.68. Besides, they also estimated the number of imported infections from Wuhan to some major cities in China. In the following, Kucharski et al. (7) fitted a stochastic transmission dynamic model with data which includes the cases in Wuhan and internationally exported cases from Wuhan, and estimated that the basic reproductive number declined from 2.35 to 1.05

With the spread of COVID-19 around the world, many scholars were also mathematically modeling the evolution trend of the epidemic in the United States, Britain, Italy, and other countries (13–23). For instance, Reiner et al. (13) used a deterministic susceptible-exposed-infectious-recovered (SEIR) compartmental framework to model the COVID-19 infections in the United States at the state level and assessed scenarios of social distancing mandates and levels of mask use. Giordano et al. (16) established a relatively comprehensive model with eight variables, which included susceptible (*S*), infected (*I*), diagnosed (*D*), ailing (*A*), recognized (*R*), threatened (*T*), healed (*H*), and extinct (*E*). The infected individuals were distinguished based on the severity of their symptoms and if they were diagnosed or not.

Unfortunately, the aforementioned models failed to consider individuals who are asymptomatic and undiagnosed in modeling the COVID-19 epidemic in the United States, and no theoretical support was provided for the sensitivity analysis of parameters. This may limit the accuracy of forecasting and the reliability of results.

To resolve this problem, this study presents a novel epidemic model, which divided the population into the Susceptible (*S*), Asymptomatic and undetected (*A*), Asymptomatic and detected (*A*_*D*_), Symptomatic and infected (*I*), recovered (*R*) and death (*D*) groups. Besides, we also use a global sensitivity analysis method to compute the sensitivity indexes of all parameters in order to provide theoretical support for parameter sensitivity and verify it by numerical simulations.

## Methods

### Mathematical Model

**Figure 1:**
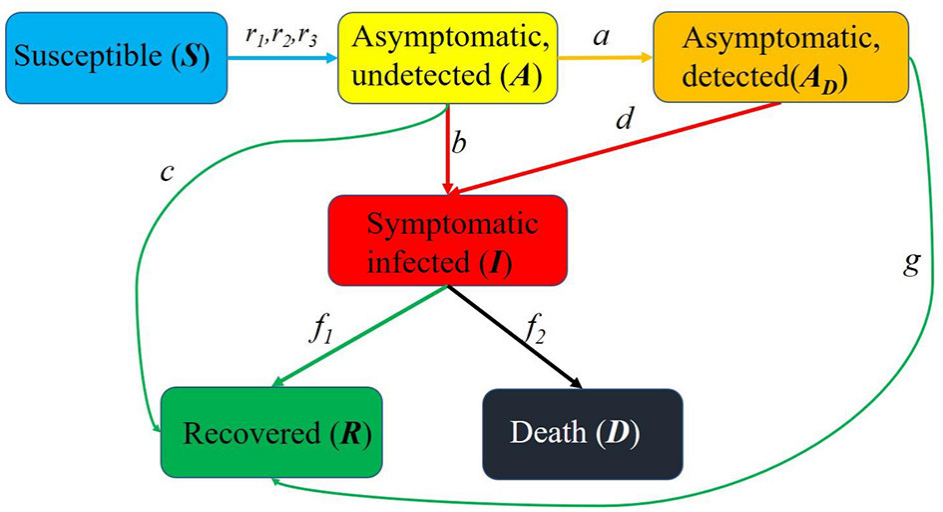
Schematic diagram of the spread of the epidemic in United States. *S*(*t*) is the susceptible, *A*(*t*) is the asymptomatic and undetected, *A*_*D*_(*t*) is the asymptomatic and detected, *I*(*t*) is the symptomatic and infected, *R*(*t*) is the recovered and *D*(*t*) is the death.

Here is an overview of the transmission mechanism of COVID-19 in the population (Figure 1):

It should be noted that the birth rate of newborns and the natural mortality rate of the population were ignored in this study.

The first subject is the susceptible population *S*(*t*). Because the birth rate of newborns was ignored, there is no source of the susceptible population, and the output includes contacts of susceptible people and asymptomatic undiagnosed people, asymptomatic diagnosed people, and symptomatic infected people. The infection rates are *r*_1_, *r*_2_, *r*_3_, respectively.

Followed by the asymptomatic untested population *A*(*t*), whose sources are contacts among susceptible people and asymptomatic undiagnosed people, asymptomatic diagnosed people, and symptomatic infected people. Then, the input includes *r*_1_*S*(*t*)*A*(*t*), *r*_2_*S*(*t*)*A*_*D*_(*t*), *r*_3_*S*(*t*)*I*(*t*), and the outputs are the asymptomatic undiagnosed people will be diagnosed as asymptomatic people with probability *a*, be diagnosed as a symptomatically infected population *b*, and with probability *c* to develop into a cured population.

The source of the asymptomatic diagnosed population *A*_*D*_(*t*) is the asymptomatic undiagnosed population that will be diagnosed as asymptomatic diagnosed population with probability *a*. The output includes the development of symptomatic infection population with probability *d* and the cure with probability *g*.

The sources of symptomatic infected population *I*(*t*) include the asymptomatic undiagnosed people with probability *b* to develop into symptomatic infected population and asymptomatic diagnosed people with probability *d* to develop into symptomatic infected population. The output includes part of the symptomatically infected people are cured with probability *f*_1_, and part of them died with probability *f*_2_.

The source of the recovered population *R*(*t*) includes three parts: first, the asymptomatic undiagnosed population is cured with probability *c*, the second is the asymptomatic diagnosed population is cured with probability *g*, and the third is the symptomatic infected population is cured with probability *f*_1_.

Without considering the natural death of the population, the source of the death population *D*(*t*) is the development of the symptomatic infection population with probability *f*_2_.

Therefore, this study proposed a mathematical model with Susceptible (*S*), Asymptomatic and undiagnosed (*A*), Asymptomatic and diagnosed (*A*_*D*_), Symptomatic and infected (*I*), recovered (*R*) and death (*D*) groups.


(1) $$\begin{array}{l} \;\frac{dS(t)}{dt}=-r_{1}S(t)A(t)-r_{2}S(t)A_{D}(t)-r_{3}S(t)I(t),\\ \;\frac{dA(t)}{dt}=r_{1}S(t)A(t)+r_{2}S(t)A_{D}(t)+r_{3}S(t)I(t)\\ \;-aA\mathrm{(t)}-bA\mathrm{(t)}-cA\mathrm{(t)}\text{, }\\ \frac{dA_{D}(t)}{dt}=aA(t)-dA_{D}(t)-gA_{D}(t),\\ \frac{\;dI(t)}{dt}=bA(t)\text{+}dA_{D}(t)-(f_{1}+f_{2})I(t),\\ \frac{\;dR(t)}{dt}=cA(t)+gA_{D}(t)+f_{1}I(t),\\ \;\frac{dD(t)}{dt}=f_{2}I(t). \end{array}$$

In the above equation (1), *S*(*t*), *A*(*t*), *A*_*D*_(*t*), *I*(*t*), *R*(*t*), *D*(*t*) represent the susceptible population, asymptomatic undiagnosed population, asymptomatic diagnosed population, symptomatic infected population, recovered population, and death population in the United States at time *t*, respectively.

### Model Parameter Estimation

We infer the model parameters based on the *baidu* data in United States from February 22, 2020 (day 1) to January 10, 2021 (day 324). The data from February 22, 2020 to December 1, 2020, which were used for the training set, are provided in Table 1 and were turned into fractions over the whole United States population (~3 ×10^8^) for simulations.

**Table 1:** The training set for model parameter estimation.

**Data**	**Symptomatic infected**	**Recovered**	**Death**	**Data**	**Symptomatic infected**	**Recovered**	**Death**
2.22	34	0	0	7.13	1,780,035	1,518,254	137,863
2.23	34	0	0	7.14	1,801,226	1,550,121	138,459
2.24	32	0	0	7.15	1,824,521	1,601,508	139,447
2.25	53	0	0	7.16	1,855,514	1,646,933	140,460
2.26	57	0	0	7.17	1,896,954	1,681,060	141,432
2.27	60	0	0	7.18	1,896,071	1,751,902	142,400
2.28	60	0	0	7.19	1,935,865	1,775,491	143,012
2.29	64	0	0	7.20	1,955,155	1,802,550	143,321
3.01	68	0	1	7.21	1,984,202	1,851,157	144,220
3.02	87	0	2	7.22	2,011,996	1,889,285	145,271
3.03	97	3	6	7.23	2,028,486	1,943,698	146,500
3.04	113	3	9	7.24	2,067,715	1,982,124	147,676
3.05	142	8	11	7.25	2,088,479	2,035,976	148,848
3.06	211	8	14	7.26	2,122,044	2,061,879	149,541
3.07	318	10	17	7.27	2,143,826	2,090,298	149,945
3.08	416	10	19	7.28	2,160,101	2,139,817	151,478
3.09	540	10	22	7.29	2172702	2190356	152729
3.10	681	10	26	7.30	2,170,004	2,246,212	153,887
3.11	969	10	31	7.31	2,213,373	2,286,492	155,746
3.12	1,269	15	38	8.01	2,240,731	2,331,327	157,184
3.13	1,187	41	36	8.02	2,256,909	2,363,229	158,039
3.14	1,581	56	41	8.03	2,281,692	2,381,407	158,457
3.15	3,381	56	62	8.04	2,265,419	2,449,120	159,386
3.16	4,483	56	90	8.05	2,281,049	2,483,903	160,502
3.17	5,741	56	97	8.06	2,284,677	2,541,859	161,895
3.18	9,089	106	150	8.07	2,299,218	2,579,191	162,975
3.19	13,924	121	205	8.08	2,325,564	2,618,203	164,377
3.20	19,217	147	260	8.09	2359059	2639927	165235
3.21	21,618	147	278	8.10	2,379,084	2,667,649	165,766
3.22	32,130	178	409	8.11	2,347,428	2,727,642	166,707
3.23	45,602	178	552	8.12	2,395,349	2,758,382	168,253
3.24	52394	178	696	8.13	2,382,457	2,813,845	169,225
3.25	63,981	378	926	8.14	2,412,378	2,844,525	170,734
3.26	81,626	680	1,201	8.15	2,430,361	2,876,080	171,568
3.27	99,207	869	1,581	8.16	2,462,639	2,904,440	172,762
3.28	118,146	961	2,010	8.17	2,476,020	2,924,268	173,187
3.29	134,578	2,661	2,436	8.18	2,466,297	2,974,788	173,804
3.30	152,816	5,595	2,956	8.19	2,475,698	3,012,244	175,429
3.31	174,534	6,043	3,606	8.20	2,470,733	3,063,412	176,628
4.01	193,231	8,434	4,542	8.21	2,483,252	3,097,040	177,652
4.02	221,830	8,861	5,648	8.22	2,502,185	3,127,665	179,489
4.03	254,139	9,445	6,889	8.23	2,520,400	3,148,165	180,295
4.04	288,583	14,997	8,496	8.24	2,536,365	3,168,960	180,720
4.05	307,819	16,848	9,458	8.25	2,522,770	3,219,333	181,479
4.06	333,281	1,9313	1,0755	8.26	2,526,445	3,257,748	182,817
4.07	359,895	21,571	12,716	8.27	2,514,400	3,315,120	183,931
4.08	387,932	23,292	14,604	8.28	2,524,487	3,350,304	185,160
4.09	421,790	25,139	16,504	8.29	2,540,826	3,376,815	186,179
4.10	452,421	27,744	18,509	8.30	2,545,832	3,409,063	186,883
4.11	480,427	29,444	20,513	8.31	2,564,034	3,425,925	187,248
4.12	505,118	32,091	22,036	9.01	2,568,607	3,458,244	187,839
4.13	526,353	36,948	23,640	9.02	2,573,697	3,498,209	188,975
4.14	546,214	28,562	25,856	9.03	2,563,781	3,548,122	190,300
4.15	564,898	48,105	28,394	9.04	2,574,039	3,575,866	191,221
4.16	583,098	57,256	34,475	9.05	2,571,360	3,637,002	192,308
4.17	612,353	60,510	37,158	9.06	2,5347,72	3,707,191	192,887
4.18	631,417	68,269	39,011	9.07	2,546,698	3,726,119	193,283
4.19	652,062	69,956	40,478	9.08	2,537,850	3,759,134	193,648
4.20	675,065	72,015	42,303	9.09	2,529,034	3,797,941	194,381
4.21	696990	82973	45343	9.10	2507170	3856749	195590
4.22	713,652	83,910	47,430	9.11	2,515,712	3,882,285	196,412
4.23	742,747	85,021	49,729	9.12	2,528,702	3,919,169	197,629
4.24	771,331	93,275	51,742	9.13	2,535,455	3,950,648	198,189
4.25	785,201	116,167	54,120	9.14	2,538,980	3,981,346	198,643
4.26	810,968	118,735	55,357	9.15	2,524,502	4,029,477	199,216
4.27	810,824	137,591	56,527	9.16	2,526,877	4,069,609	200,667
4.28	831,100	140,138	58,640	9.17	2,516,348	4,120,577	201,631
4.29	850,146	145,320	61,180	9.18	2,521,404	4,156,472	202,306
4.30	877,117	151,774	63,765	9.19	2,536,091	4,192,963	203,274
5.01	899,592	160,173	65,540	9.20	2,544,563	4,223,996	203,881
5.02	927,734	161,782	67,228	9.21	2,554,930	4,251,943	204,165
5.03	938,453	178,219	68,495	9.22	2,547,459	4,301,523	204,801
5.04	955,872	184,354	69,476	9.23	2,540,742	4,360,093	205,864
5.05	963,387	199,151	72,054	9.24	2,541,219	4,400,872	206,895
5.06	977,250	205,268	74,121	9.25	2,548,693	4,440,485	207,794
5.07	996,657	215,580	76,791	9.26	2,558,753	4,483,950	208,625
5.08	1,018,180	222,008	78,498	9.27	2,560,645	4,524,760	209,238
5.09	1,029,928	232,869	79,926	9.28	2,544,348	4,571,265	209,502
5.10	1,043,738	240,853	80,717	9.29	2,544,223	4,611,282	209,922
5.11	1,039,925	260,188	81,552	9.30	2,551,485	4,651,017	211,098
5.12	1,042,748	280,509	83,262	10.01	2,533,872	4,711,997	211,988
5.13	1,034,803	307,755	85,029	10.02	2,544,436	4,750,176	212,912
5.14	1,050,367	316,244	86,770	10.03	2,571,824	4,779,402	213,684
5.15	1,071,318	321,348	88,309	10.04	2,564,550	4,828,654	214,341
5.16	1,076,667	337,563	89,454	10.05	2,567,993	4,862,023	214,697
5.17	1,090,398	344,805	90,931	10.06	2,565,240	4,906,808	215,221
5.18	1,094,544	347,225	91,092	10.07	2,564,726	4,950,141	216,064
5.19	1,103,798	359,137	92,198	10.08	2,573,418	4,997,380	217,081
5.20	1,118,652	361,419	93,707	10.09	2,598,022	5,026,952	217,857
5.21	1,130,935	371,077	95,118	10.10	2,623,925	5,066,257	218,855
5.22	1,147,627	383,099	96,683	10.11	2,645,333	5,092,941	219,341
5.23	1,149,562	403,315	97,800	10.12	2,642,681	5,138,374	219,797
5.24	1,126,524	447,211	98,792	10.13	2,632,448	5,197,125	220,281
5.25	1,138,597	451,749	99,381	10.14	2,646,551	5,238,565	221,147
5.26	1,143,620	467,962	99,987	10.15	2,652,405	5,290,510	222,092
5.27	1,149,449	480,321	100,825	10.16	2,678,060	5,329,151	222,973
5.28	1,157,822	490,262	102,293	10.17	2,679,683	5,402,456	223,885
5.29	1,169,423	499,768	103,452	10.18	2,688,666	5,438,389	224,389
5.30	1,173,579	519,736	104,634	10.19	2,705,539	5,463,410	224,824
5.31	1,180,203	536,234	105,680	10.20	2,729,188	5,513,584	225,451
6.01	1,135,791	600,150	106,302	10.21	2,753,012	5,549,360	226,360
6.02	1,140,025	615,719	107,135	10.22	2,755,002	5,612,505	227,516
6.03	1,132,803	6,46,614	1,08,291	10.23	2,783,624	5,662,998	228,577
6.04	1,109,287	689,282	109,271	10.24	2,835,194	5,704,352	2,29,551
6.05	1,105,258	712,437	110,331	10.25	2,866,520	5,742,963	230,126
6.06	1,123,286	738,998	111,599	10.26	2,886,403	5,781,451	230,556
6.07	1,130,819	752,848	112,187	10.27	2,902,462	5,842,665	231,308
6.08	1,137,669	761,736	112,596	10.28	2936080	5885393	232305
6.09	1,143,360	773,696	113,267	10.29	2,962,886	5,940,558	233,340
6.10	1,149,468	788,969	114,379	10.30	3,005,844	5,986,309	234,405
6.11	1,148,427	808,556	115,291	10.31	3,070,434	6,030,186	235,453
6.12	1,160,893	8,173,37	116,138	11.01	3,111,594	6,066,893	236,154
6.13	1,163,821	842,329	116,952	11.02	3,140,841	6,109,683	236,564
6.14	1,177,999	854,659	117,587	11.03	3,163,771	6,173,165	237,068
6.15	1,178,703	870,080	117,920	11.04	3,226,079	6,237,659	238,746
6.16	1,178,643	891,068	118,487	11.05	3,272,622	6,294,444	239,894
6.17	1,193,135	903,176	119,269	11.06	3,353,866	6,342,279	241,126
6.18	1,203,727	919,108	120,079	11.07	3,436,331	6,392,425	242,339
6.19	1,223,019	931,355	120,844	11.08	3,505,429	6,442,590	243,316
6.20	1,225,856	9,56,316	121,520	11.09	3,570,006	6,484,054	243,807
6.21	1,245,784	974,746	122,067	11.10	3,634,241	6,5545,26	244,589
6.22	1,260,697	980,836	122,292	11.11	3,730,426	6,603,478	246,034
6.23	1,270,015	1,003,322	122,764	11.12	3,825,920	6,651,545	247,537
6.24	1,282,480	1,020,499	123,521	11.13	3,910,111	6,729,527	248,686
6.25	1,309,829	1,040,711	124,422	11.14	4,037,659	6,790,898	250,105
6.26	1,332,344	1,052,529	126,911	11.15	4,092,920	6,891,461	251,285
6.27	1,374,303	1,069,342	127,803	11.16	4,190,156	6,939,835	251,965
6.28	1,405,391	1,081,793	128,233	11.17	4,269,508	7,023,230	252,792
6.29	1,427,320	1,093,951	128,503	11.18	4,354,568	7,090,336	254,329
6.30	1,443,976	1,122,678	129,031	11.19	4,467,547	7,171,883	256,609
7.01	1,470,302	1,143,923	130,345	11.20	4,586,626	7,248,771	258,655
7.02	1,498,317	1,168,436	130,984	11.21	4,712,918	7,320,373	260,479
7.03	1,532,403	1,191,892	131,666	11.22	4,796,348	7,405,265	261,885
7.04	1,539,182	1,237,767	132,174	11.23	4,882,471	7,453,661	262,757
7.05	1,555,518	1,260,695	132,374	11.24	4,968,719	7,553,556	263,899
7.06	1,572,048	1,291,315	132,664	11.25	5,070,635	7,641,913	266,285
7.07	1,595,611	1,326,669	133,268	11.26	5,072,840	7,809,461	268,439
7.08	1,626,369	1,355,898	134,163	11.27	5,250,361	7,945,585	271,038
7.09	1,646,372	1,393,363	135,189	11.28	5,299,020	8,041,239	272,253
7.10	1,679,417	1,426,645	136,024	11.29	5,370,261	8,107,270	2,73,077
7.11	1,713,521	1,461,374	136,949	11.30	5,422,315	8,223,391	274,332
7.12	1,74,1831	1,501,866	137,577	12.01	5,428,909	8,230,001	274,743

The estimated parameter values are based on the gathered data which are the number of currently infected individuals *I*(*t*), the number of diagnosed individuals who recovered *R*(*t*) and the number of death *D*(*t*) because of SARS-CoV-2 virus.

The nonlinear least square (NLS) method is regarded as the most basic way to estimate unknown parameters for ordinary differential equation model and facilitate to implement algorithm (24). Therefore, the NLS method was adopted to find the parameters that locally minimize the sum of the squares of the errors. During the course of the simulation, the parameters have been updated based on the successive measures at different stages.

### NLS Method

Firstly, let P = (*r*_1_, *r*_2_, *r*_3_, *a, b, c, d, g, f*_1_, *f*_2_), and *I*(*t*, P), *R*(*t*, P) and *D*(*t*, P) are the numerical solution of *I*(*t*), *R*(*t*) and *D*(*t*). Then, collect the data of currently infected individuals *I*_0_, *I*_1_, *I*_2_, …, *I*_*n*−1_, the number of diagnosed individuals who recovered *R*_0_, *R*_1_, *R*_2_, …, *R*_*n*−1_ and the number of death *D*_0_, *D*_1_, *D*_2_, …, *D*_*n*−1_. The initial values of six kinds of individuals are *S*_0_, *A*_0_, *A*_*D*0_, *I*_0_, *R*_0_, *D*_0_.

We assumed that *I*(*t*_*i*_, P), *R*(*t*_*i*_, P) and *D*(*t*_*i*_, P) are the numerical solution at *t*_*i*_ and the parameter vector is P = (*r*_1_, *r*_2_, *r*_3_, *a, b, c, d, g, f*_1_, *f*_2_). Next, we need to find the best parameter vector $\tilde{\text{P}}=\left({\tilde{r}}_{1},{\tilde{r}}_{2},{\tilde{r}}_{3},ã,\tilde{b},\tilde{c},\tilde{d},\tilde{g},{\tilde{f}}_{1},{\tilde{f}}_{2}\right)$ to minimize the following equation:


$$\begin{array}{l} \text{sum}=\overset{n-1}{\underset{i=0}{\sum }}\sqrt{{\left(I\left(t_{i},\text{P}\right)-I_{i}\right)}^{2}+{\left(R\left(t_{i},\text{P}\right)-R_{i}\right)}^{2}+{\left(D\left(t_{i},\text{P}\right)-D_{i}\right)}^{2}} \end{array}$$

Furthermore, based on previous literature, give the initial value of parameter vector ${\text{P}}^{0}=\left({r_{1}}^{0},{r_{2}}^{0},{r_{3}}^{0},a^{0},b^{0},c^{0},d^{0},g^{0},{f_{1}}^{0},{f_{2}}^{0}\right)$ and set the bound. Use the *fmincon* function to estimate the approximate range of each parameter and the estimated parameters were regarded as new initial values. Lastly, the *lsqnonlin* function was employed to achieve the best fitting effect and get the optimal parameters.

### Parameter Sensitivity Analysis

Similar to our previous research (25), extended Fourier Amplitude Sensitivity Test (EFAST) was adopted to search the sensitive parameters. The first order sensitivity index (*S*_*i*_) and full order sensitivity index (*S*_*Ti*_) are calculated by the following equations:


(2) $$\begin{array}{l} S_{i}=\frac{V_{i}}{\text{Var}\left(Y\right)} \end{array}$$

(3) $$\begin{array}{l} S_{Ti}=\frac{V_{i}+V_{ij}+\ldots +V_{ij\ldots k}}{\text{Var}\left(Y\right)} \end{array}$$

Based on reference (26), the sensitivity value (*S*_*Ti*_) larger than 0.5 was defined as a sensitive parameter; otherwise, it was defined as a non-sensitive parameter.

## Results

### Basic Reproduction Number and Attack Rates

The basic reproduction number reflects the size of the virus transmission capacity. The larger the basic reproduction number, the stronger the virus transmission ability; the smaller the basic reproduction number, the weaker the transmission ability. Therefore, the study on basic reproduction number is very necessary.

Based on equation (1), the equilibrium $\left(S^{*},A^{*},A_{D}^{*},I^{*},R^{*},D^{*}\right)=\left(S^{*},0,0,0,R^{*},D^{*}\right)$ was calculated firstly, and the Jacobian matrix around the equilibrium is:


$$\begin{array}{l} Jac=\\ \;\left[\begin{array}{cccccc} -r_{1}A^{\text{*}}-r_{2}A_{D}^{*}-r_{3}I^{\text{*}} & -r_{1}S^{\text{*}} & -r_{2}S^{\text{*}} & -r_{3}S^{\text{*}} & 0 & 0\\ r_{1}A^{\text{*}}+r_{2}A_{D}^{*}+r_{3}I^{\text{*}} & r_{1}S^{\text{*}}-a-b-c & r_{2}S^{\text{*}} & r_{3}S^{\text{*}} & 0 & 0\\ 0 & a & -d-g & 0 & 0 & 0\\ 0 & b & d & -f_{1}-f_{2} & 0 & 0\\ 0 & c & g & f_{1} & 0 & 0\\ 0 & 0 & 0 & f_{2} & 0 & 0 \end{array}\right] \end{array}$$

The corresponding Jacobian determinant is:


$$\begin{array}{l} |Jac|=\\ \;\left|\begin{array}{cccccc} \lambda \text{+}r_{1}A^{\text{*}}+r_{2}A_{D}^{*}+r_{3}I^{\text{*}} & r_{1}S^{\text{*}} & r_{2}S^{\text{*}} & r_{3}S^{\text{*}} & 0 & 0\\ -r_{1}A^{\text{*}}-r_{2}A_{D}^{*}-r_{3}I^{\text{*}} & \lambda -r_{1}S^{\text{*}}+a+b+c & -r_{2}S^{\text{*}} & -r_{3}S^{\text{*}} & 0 & 0\\ 0 & -a & \lambda +d+g & 0 & 0 & 0\\ 0 & -b & -d & \lambda +f_{1}+f_{2} & 0 & 0\\ 0 & -c & -g & -f_{1} & \lambda & 0\\ 0 & 0 & 0 & -f_{2} & 0 & \lambda \end{array}\right| \end{array}$$

$$\begin{array}{l} =\lambda^{3}\left(\lambda +f_{1}+f_{2}\right)\left(\lambda +a+b+c\right)\left(\lambda +d+g\right)\\ \;-\lambda^{3}S^{\text{*}}[r_{1}\left(\lambda +f_{1}+f_{2}\right)\left(\lambda +d+g\right)\text{+}adr_{3}\\ \;+ar_{2}\left(\lambda +f_{1}+f_{2}\right)+br_{3}\left(\lambda +d+g\right)] \end{array}$$

Let


(4) $$\begin{array}{l} p\left(\lambda \right)=D\left(\lambda \right)-S^{*}N\left(\lambda \right) \end{array}$$

where


$$\begin{array}{l} \begin{array}{lll} D(\lambda ) & = & \left(\lambda +f_{1}+f_{2})(\lambda +a+b+c)(\lambda +d+g\right)\\ N(\lambda ) & = & r_{1}(\lambda +f_{1}+f_{2})(\lambda +d+g)\text{+}adr_{3}\text{+}ar_{2}(\lambda +f_{1}+f_{2})\\ \; & \; & +br_{3}(\lambda +d+g). \end{array} \end{array}$$

Therefore, we get *G*(λ) is as follows:


$$\begin{array}{l} G\left(\lambda \right)=\frac{N\left(\lambda \right)}{D\left(\lambda \right)}=\\ \;\frac{r_{1}\left(\lambda +f_{1}+f_{2}\right)\left(\lambda +d+g\right)\text{+}adr_{3}\text{+}ar_{2}\left(\lambda +f_{1}+f_{2}\right)+br_{3}\left(\lambda +d+g\right)}{\left(\lambda +f_{1}+f_{2}\right)\left(\lambda +a+b+c\right)\left(\lambda +d+g\right)} \end{array}$$

The system is positive and $||G\left(\lambda \right)|{|}_{\infty }=G\left(0\right)=\frac{N\left(0\right)}{D\left(0\right)}$, where ||*G*(λ)||_∞_ is the *H*_∞_ norm of *G*(λ).

Based on Hurwitz criterion (27) and conference (16), all roots in the left-hand plane if, and only, if S^*^<*G*(0). Therefore, the basic reproduction number is:


(5) $$\begin{array}{l} R_{0}=\frac{1}{S^{*}}=G\left(0\right)\\ =\frac{r_{1}\left(f_{1}+f_{2}\right)\left(d+g\right)\text{+}adr_{3}\text{+}ar_{2}\left(f_{1}+f_{2}\right)+br_{3}\left(d+g\right)}{\left(f_{1}+f_{2}\right)\left(a+b+c\right)\left(d+g\right)}\; \end{array}$$

The attack rate denotes the proportion of all infected individuals to the total population, and the infected individuals include the asymptomatic and undetected, the asymptomatic and detected, and the symptomatic and infected. The method for calculation of the attack rate is:


(6) $$\begin{array}{l} attack\;rate=\frac{A\left(t\right)+A_{D}\left(t\right)+I\left(t\right)}{S\left(t\right)+A\left(t\right)+A_{D}\left(t\right)+I\left(t\right)+R\left(t\right)+D\left(t\right)} \end{array}$$

### Parameter Estimation

Since the development trend of the epidemic varies with the different control strategies adopted by countries, the parameters of model were estimated by piecewise fitting method in this study. Based on *Baidu* data and the above NLS algorithm, the model parameters obtained by piecewise fitting are as follows:

The parameters from days 1 to 50 are:

*r*_1_ = 0.049477, *r*_2_ = 1.3392*e*−05, *r*_3_ = 0.27981, *a* = 0.23686, *b* = 0.71424, *c* = 0.0233352, *d* = 0.79452, *g* = 0.0040538, *f*_1_ = 0.0061802, *f*_2_ = 0.009, and the basic reproduction number is *R*_0_ = 6.0064.

The parameters from day 51 to day 100 are:

*r*_1_ = 0.049477, *r*_2_ = 1.3392*e*−05, *r*_3_ = 0.27981, *a* = 0.13686, *b* = 0.009424, *c* = 0.007352, *d* = 0.007452, *g* = 0.0018538, *f*_1_ = 0.0061802, *f*_2_ = 0.003, and the basic reproduction number is *R*_0_ = 6.2908.

The parameters from days 101 to 200 are:

*r*_1_ = 0.049477, *r*_2_ = 1.3392*e*−03, *r*_3_ = 0.0131, *a* = 0.13686, *b* = 0.008424, *c* = 0.0053352, *d* = 0.003052, *g* = 0.0018538, *f*_1_ = 0.0061802, *f*_2_ = 0.0047, and the basic reproduction number is *R*_0_ = 1.8003.

The parameters from days 201 to 250 are:

*r*_1_ = 0.049477, *r*_2_ = 1.3392*e*−03, *r*_3_ = 0.0131, *a* = 0.13686, *b* = 0.004424, *c* = 0.0053352, *d* = 0.003052, *g* = 0.003538, *f*_1_ = 0.006, *f*_2_ = 0.003, and the basic reproduction number is *R*_0_ = 1.4888.

The parameters from day 251 to day 324 are:

*r*_1_ = 0.049477, *r*_2_ = 1.3392*e*−03, *r*_3_ = 0.0281, *a* = 0.13686, *b* = 0.00424, *c* = 0.0123352, *d* = 0.0138, *g* = 0.00538, *f*_1_ = 0.0062, *f*_2_ = 0.0032, and the basic reproduction number is *R*_0_ = 3.2698.

### Simulation Results and Predictions

**Figure 2:**
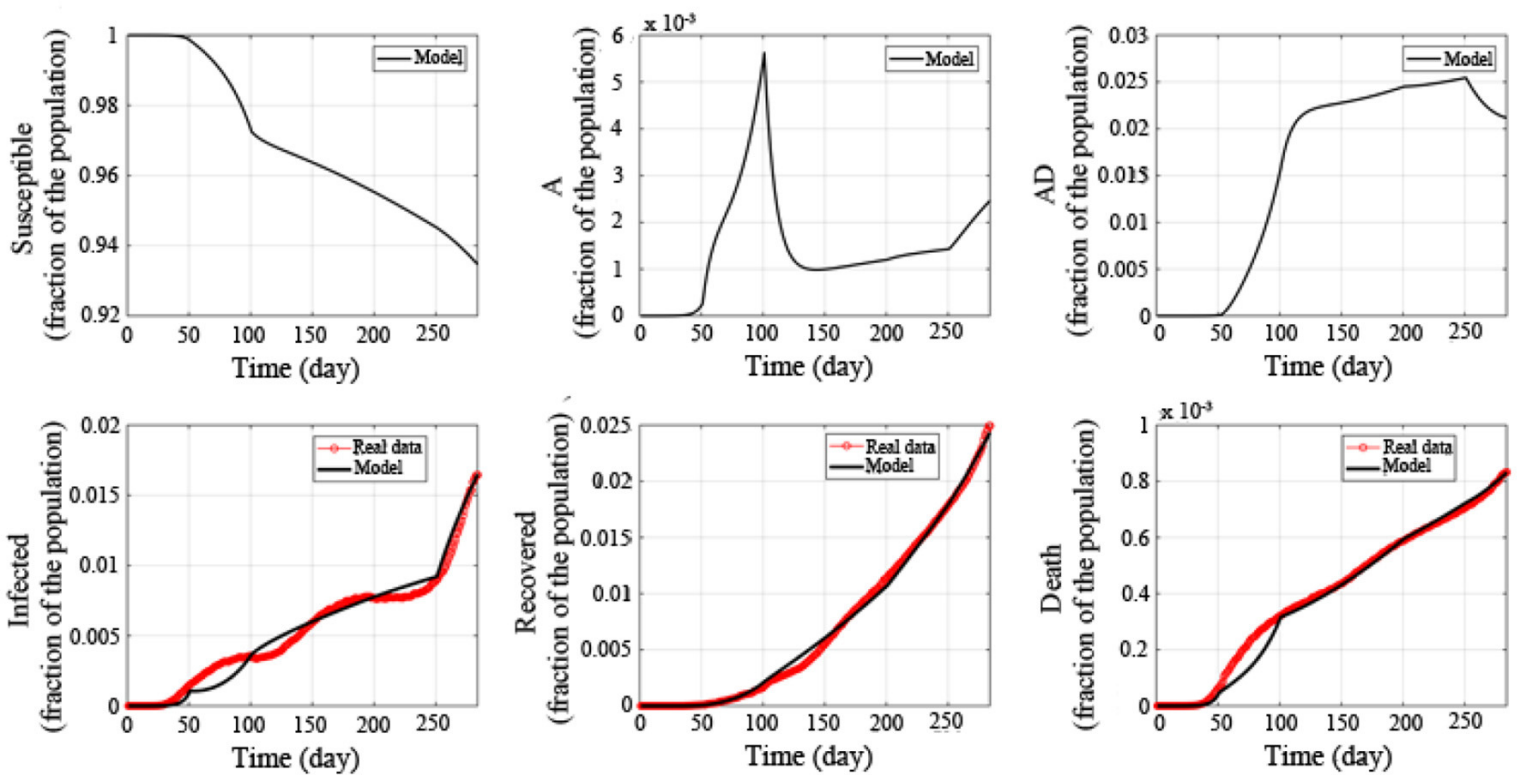
Fitted epidemic evolution by the model based on the available data about the coronavirus disease-2019 (COVID-19) outbreak in the United States.

Based on the parameter estimation of the above five stages, the fitting results are obtained, as shown in Figure 2 (from February 22, 2020 to December 1, 2020, 284 days in total). The black curve represents the simulation result of the mathematical model, and the red curves are the collected data of the current infected, recovered and death, respectively. The correlation between the number of current infected simulated by the mathematical model and the collected was 98.85%, the correlation of the recovered was 99.84%, and the correlation of the death was 99.54%.

In addition, we have also calculated the basic reproduction number in 5 stages. The basic reproduction number in the first stage is *R*_0_ = 6.0064, and the basic reproduction number in the second stage is *R*_0_ = 6.2908, indicating that the transmission capacity of the epidemic is very strong in the first two stages. If it is not controlled, it will quickly spread throughout the United States. In the third stage, the basic reproduction number is *R*_0_ = 1.4888. Compared with the first two stages, the transmission capacity of COVID-19 has been significantly reduced. In the fourth stage, the basic reproduction number further drops to *R*_0_ = 1.4888, indicating that with joint efforts of the US government and the people, the spread of the epidemic has further declined. In the fifth stage, the basic reproduction number has risen again to *R*_0_ = 3.2698, indicating that the transmission capacity of COVID-19 has increased, and that it is necessary to take measures to prevent and control it.

Next, in order to verify the accuracy of the model established in this research, we continued to collect data from December 2, 2020 to January 10, 2021 (40 days in total) (marked with blue curves). The data are provided in Table 2.

**Table 2:** Validation set for verifying the accuracy of the model.

**Data**	**Symptomatic infected**	**Recovered**	**Death**	**Data**	**Symptomatic infected**	**Recovered**	**Death**
12.02	5,508,475	8,345,995	277,396	12.22	7,359,922	10,807,172	327,171
12.03	5,584,329	8,468,702	280,210	12.23	7,411,247	10,949,574	330,921
12.04	5,714,256	8,570,636	283,300	12.24	7,498,444	11,104,857	334,415
12.05	5,835,097	8,663,942	285,786	12.25	7,560,642	11,219,489	337,081
12.06	5,917,474	8,790,495	287,894	12.26	7,618,560	11,260,932	338,324
12.07	6,021,199	8,859,465	288,984	12.27	7,736,834	11,495,875	341,138
12.08	6,098,173	8,994,191	291,016	12.28	7,741,715	11,696,727	343,182
12.09	6,220,791	9,095,080	293,739	12.29	7,758,983	11,701,029	343,593
12.10	6,313,153	9,235,316	297,173	12.30	7,795,238	11,848,630	346,955
12.11	6,433,334	9,340,223	300,272	12.31	7,881,939	12,004,898	351,127
12.12	6,493,377	9,511,911	302,904	1.01	7,978,440	12,129,680	354,381
12.13	6,612,582	9,645,924	305,144	1.02	8,083,344	12,179,238	356,450
12.14	6,718,324	9,727,555	306,529	1.03	8,195,127	12,364,189	358,745
12.15	6,771,179	9,879,331	308,335	1.04	8,319,267	12,438,638	360,151
12.16	6,833,466	10,015,012	311,316	1.05	8,262,393	12,740,254	362,538
12.17	6,923,404	10,176,485	314,991	1.06	8,376,364	12,867,806	366,252
12.18	7,040,310	10,296,261	318,413	1.07	8,472,281	13,027,453	370,151
12.19	7,178,903	10,399,339	321,025	1.08	8,642,504	13,149,021	374,624
12.20	7,215,998	10,546,751	323,466	1.09	8,851,167	13,262,863	378,559
12.21	7,337,319	10,623,101	324,915	1.10	89,37,419	13,395,752	381,557

**Figure 3:**
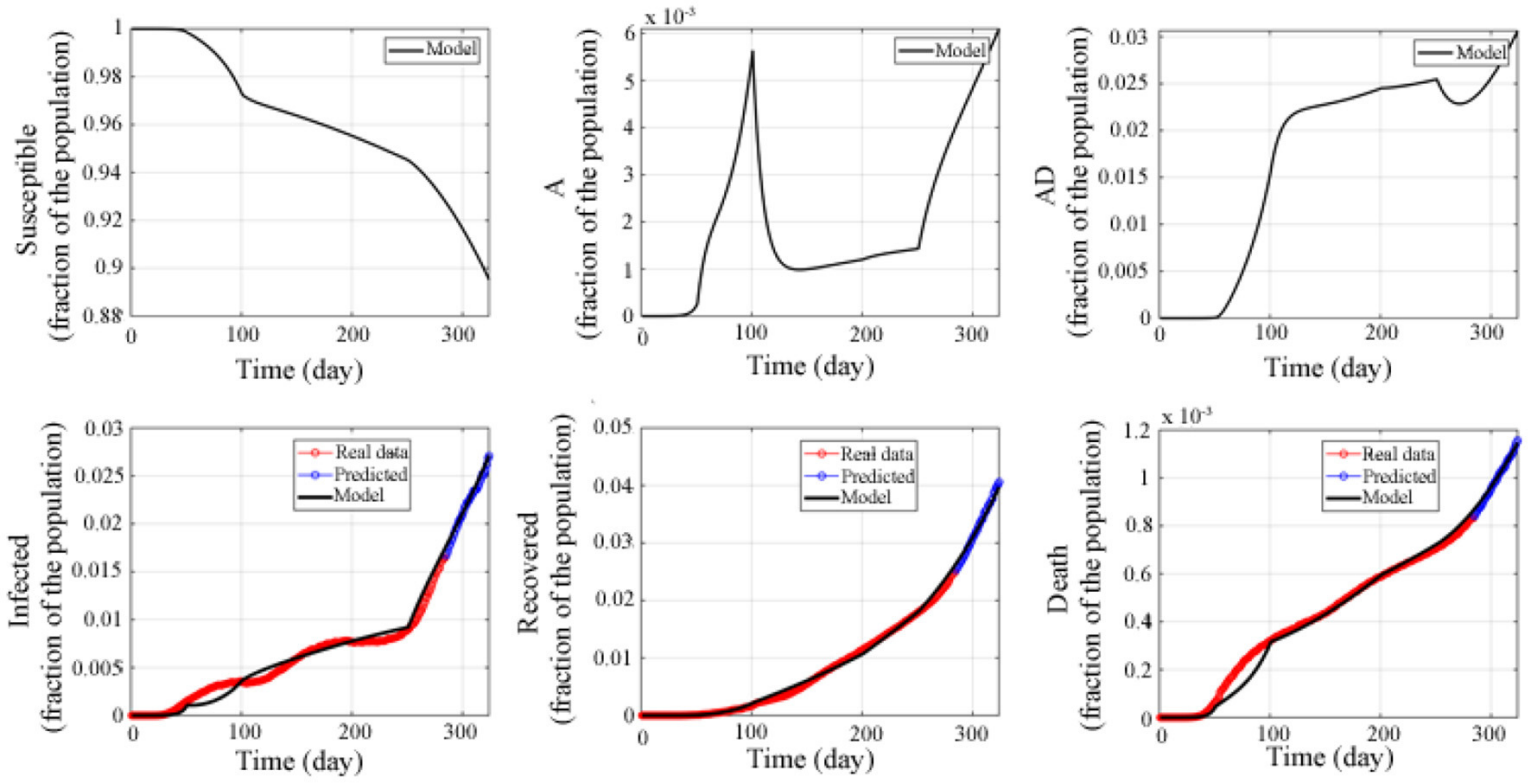
Fitted and predicted epidemic evolution. Epidemic evolution predicted by the model based on the available data about the COVID-19 outbreak in the United States.

The red curves are from February 22, 2020 to December 1, 2020, a total of 284 days, and the black curve is the simulation result of the model, as shown in Figure 3. We use the following equation to calculate the accuracy of model prediction:


(7) $$\begin{array}{l} accuracy\;rate=\left(1-\frac{1}{n}\cdot \frac{\overset{n}{\underset{i=1}{\sum }}|x_{i}-y_{i}|}{x_{i}}\right)\times 100\% \end{array}$$

where *x*_*i*_ is the real value, *y*_*i*_ is the forecasting value, and *n* is the number of all data that needs to be forecast.

The results show that the prediction accuracy rate of the current infected population *I*(*t*) is 98.33%, the prediction accuracy rate of the recovered population *R*(*t*) is 98.2%, and the prediction accuracy rate of the death population *D*(*t*) is 98.96%. This indicates that the model established in this study has high accuracy, and that the model can accurately predict the trend of the epidemic in the United States, and provides a guarantee for the following applied research.

**Figure 4:**
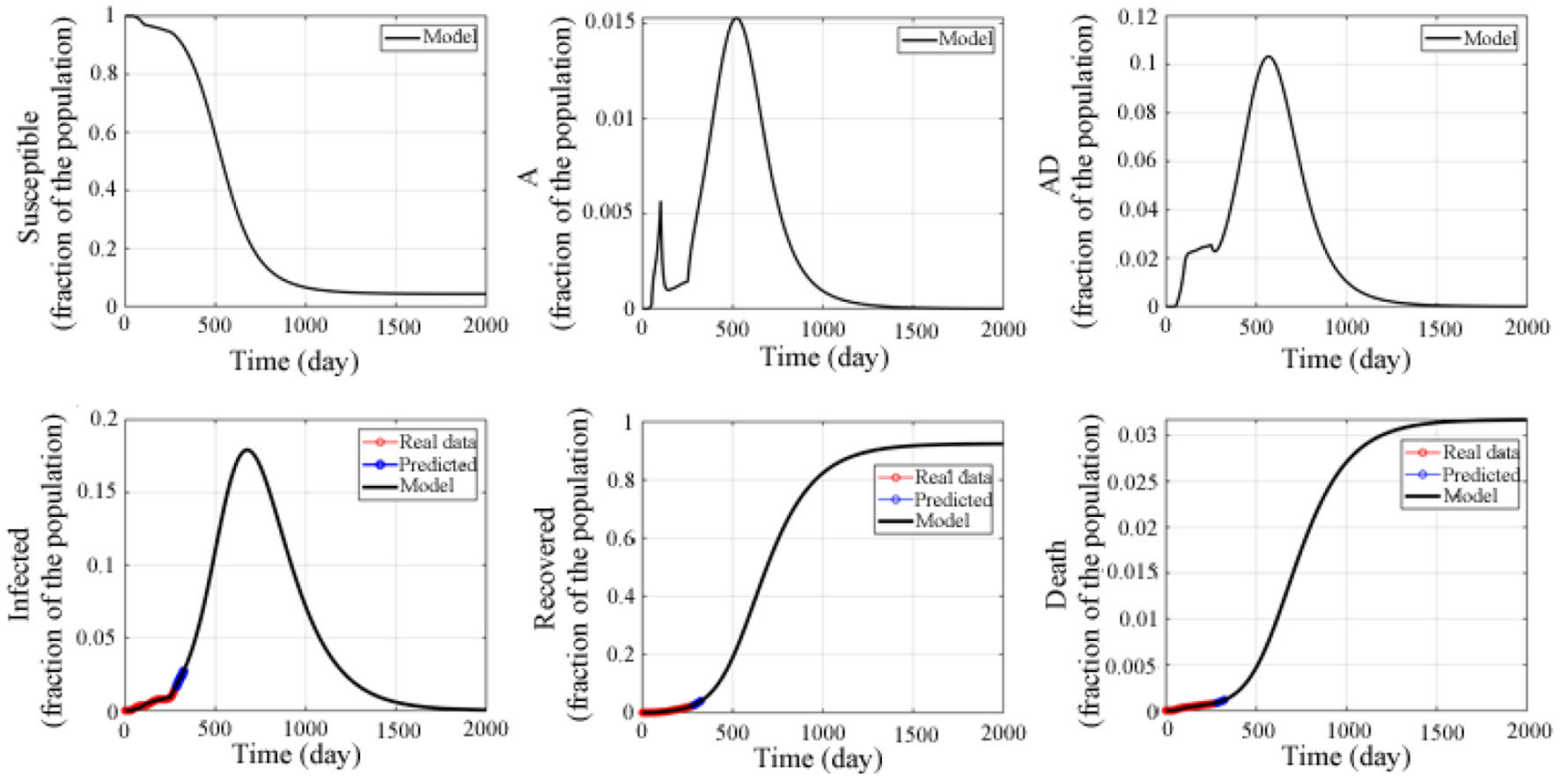
Predicted epidemic evolution for 2,000 days. Epidemic evolution predicted by the model based on the available data about the COVID-19 outbreak in the United States.

Therefore, without taking into account the vaccine and the recovery of the survivors, we used the above model to make a long-term prediction of the epidemic in the United States (as shown in Figure 4). The prediction results showed that the susceptible population continued to decline, reaching the lowest level around the 1,560th day (4.45% of the total population) and continued until the end. The asymptomatic undiagnosed population reached a peak on the 518th day (1.53% of the total population), and then gradually decreased, dropping to 0% on the 1367th day. The asymptomatic diagnosed population reached the peak on the 570th day (10.32% of the total number), and gradually decreased, and the dropped to 0% on the 1,440th day. The infected population reached a peak around the 679th day (17.92% of the total number), gradually decreased, and then dropped to 0% around the 1,852th day. The number of recovered people was increasing, reaching a peak around the 1,533th day (91.87% of the total number), and was stable at this value. The number of deaths also increased gradually, reaching a peak around the 1,619th day (3.16% of the total number), and remained stable.

**Figure 5:**
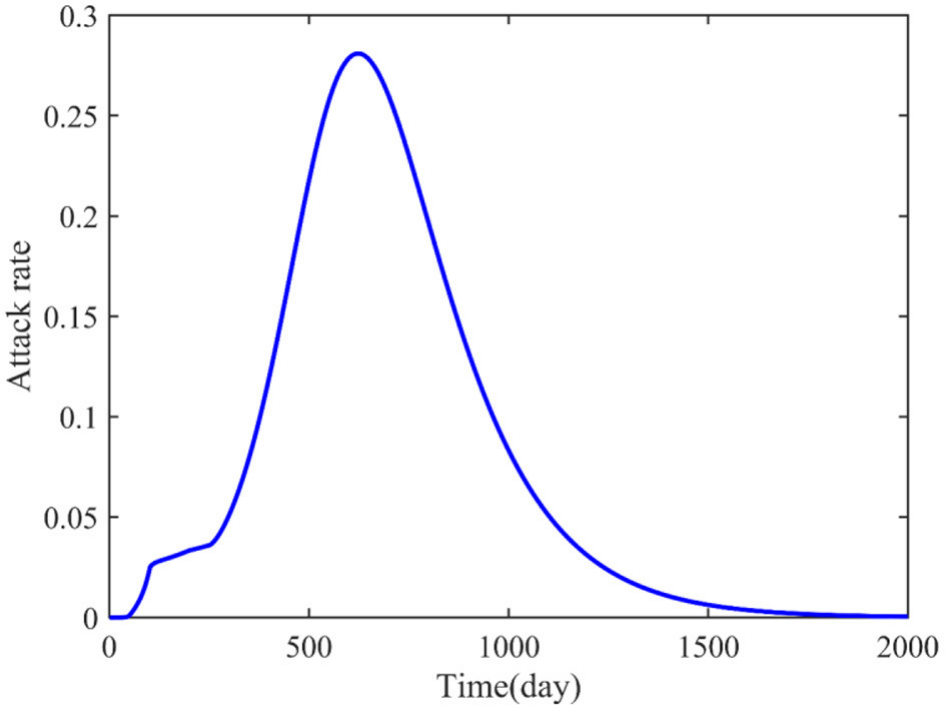
Predicted attack rate for 2,000 days.

In addition, the attack rate was also studied and shown in Figure 5. The numerical simulation result indicated that the attacked individuals increased from the beginning and reached a peak around the 624th day, and that the maximum attack rate was 0.281, which is different from the asymptomatic and undetected, the asymptomatic and detected, and the symptomatic and infected.

### Parameter Sensitivity Analysis

Next, parameter sensitivity analysis was performed to screen the factors that significantly affected the development of the epidemic. In order to overcome the influence of coupling between parameters on the results, we adopted a robust, highly calculated, and low-sample required global sensitivity analysis method, the Extended Fourier Amplitude Sensitivity Test (EFAST) method.

**Figure 6:**
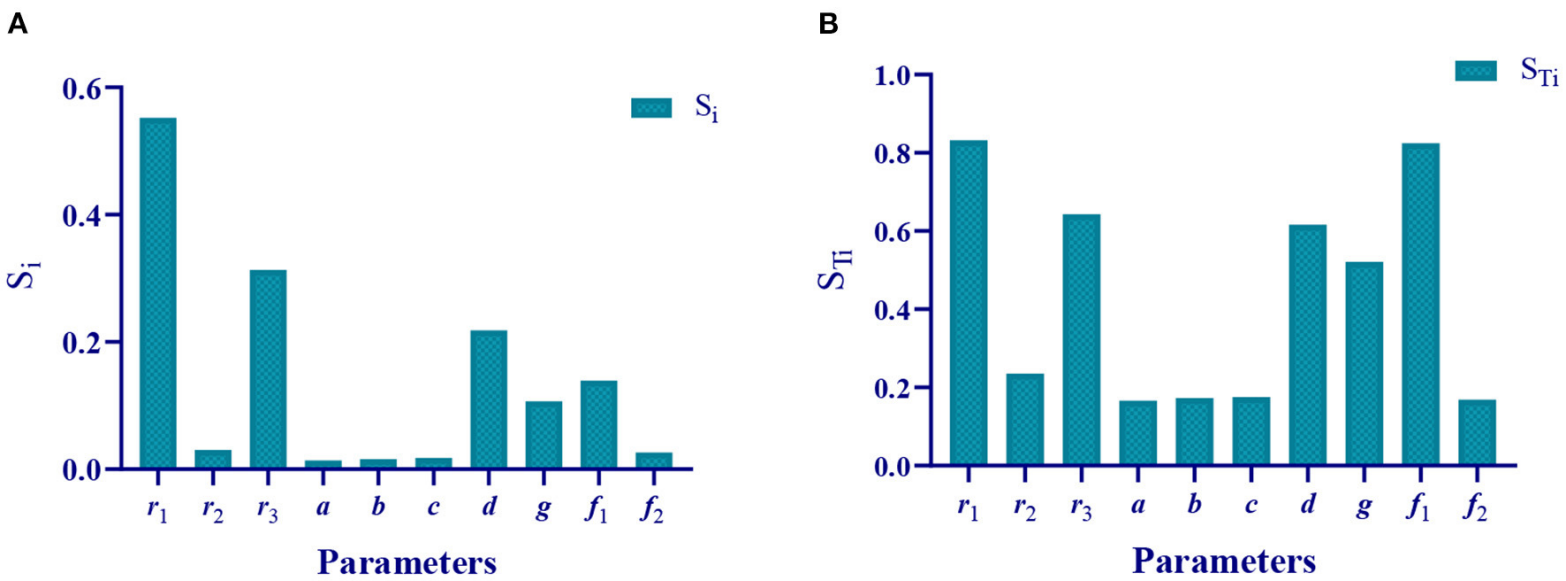
Sensitivity indexes of the 10 parameters. **(A)** First-order sensitivity index of the 10 parameters. **(B)** Full-order sensitivity index of the 10 parameters.

Based on the method of global sensitivity analysis, we obtained the first-order and full-order sensitivity indexes of 10 parameters in the mathematical model (Table 3 and Figures 6A,B). Based on reference, the sensitivity value (*S*_*Ti*_) higher than 0.5 was defined as a sensitive parameter; otherwise, it was defined as a non-sensitive parameter (26).

**Table 3:** First-order and full-order sensitivity indexes of the 10 parameters.

	***r_**1**_***	***r_**2**_***	***r_**3**_***	***a***	***b***	***c***	***d***	***g***	***f_**1**_***	***f_**2**_***
S_i_	0.5521	0.0305	0.3133	0.0136	0.0158	0.0181	0.2181	0.1066	0.1393	0.0261
S_Ti_	0.8317	0.2353	0.6427	0.1664	0.1731	0.1757	0.6163	0.5210	0.8247	0.1690

Therefore, we theoretically analyzed the parameters that affect the epidemic. The sensitivity parameters are *r*_1_ = 0.8317 (the infection rate of asymptomatic undiagnosed population to susceptible population), *r*_3_ = 0.6427 (the infection rate of symptomatic infection population to susceptible population), *d* = 0.6163 (probability of asymptomatically diagnosed population turning into symptomatically infected population), *g* = 0.521 (recovered rate of asymptomatic diagnosed population), and *f*_1_ = 0.8247 (recovered rate of symptomatically infected population), non-sensitive parameters are *r*_2_ = 0.2353 (the infection rate of the asymptomatic diagnosed population to the susceptible population), *a* = 0.1664 (the probability of the asymptomatic undiagnosed population becoming asymptomatic diagnosed population), *b* = 0.1731 (the probability of asymptomatic undiagnosed population becoming symptomatically infected), *c* = 0.1757 (the recovered rate of asymptomatic undiagnosed population), and *f*_2_ = 0.169 (the death rate of symptomatic infection).

### Verified Results of Parameter Sensitivity Analysis by Numerical Simulation

In the following, we used numerical simulation method to verify the correctness of the above global sensitivity analysis results. That is, the gradient change of parameters is used to study the impact of the output results of the susceptible population, the asymptomatic undiagnosed population, the asymptomatic diagnosed population, the symptomatic infected population, the recovered population, and the death population. For example, the initial value of parameter *r*_1_ is 0.05, and it increases to 0.1 in steps of 0.01 to study the dynamics trend of each type of population.

Numerical simulation results showed that when parameter *r*_1_ increases from 0.05 to 0.1 in steps of 0.01, the number of susceptible populations is greatly reduced, and that the number of asymptomatic undiagnosed population, asymptomatic diagnosed population, symptomatic infected population, recovered population, and death population all increases significantly with increase in *r*_1_ (as shown in Supplementary Figure 1). This is consistent with the sensitivity of *r*_1_, calculated by our theory, of 0.8317 (Table 3). Therefore, the parameter *r*_1_ is a sensitive parameter that affects the epidemic.

When parameter *r*_2_ increases from 0.0013 to 0.0018 in steps of 0.0001, the number of susceptible people, asymptomatic undiagnosed population, asymptomatic diagnosed population, symptomatic infected population, recovered population, and death population has no significant changes (Supplementary Figure 2). This is consistent with the sensitivity of parameter *r*_2_ of 0.2353 (Table 3), indicating that parameter *r*_2_ is an insensitive parameter, and that the change in parameter *r*_2_ has no significant effect on the epidemic.

When parameter *r*_3_ increased from 0.016 to 0.021 in steps of 0.001, the number of susceptible populations significantly reduced, and the number of asymptomatic undiagnosed population, asymptomatic diagnosed population, symptomatic infected population, recovered population, and death population all increased significantly with the increase in *r*_3_ (Supplementary Figure 3). This is consistent with the sensitivity of *r*_3_ of.6427 (Table 3). Therefore, parameter *r*_3_ is a sensitive parameter that affects the epidemic. The sensitivity of parameter *r*_3_ (0.6427) is lower than that of parameter *r*_3_ (0.8317). The numerical simulation results also verify that the change in parameter *r*_1_ has a greater impact on the epidemic than parameter *r*_3_, which preliminarily proves that the parameter sensitivity obtained by our theoretical calculation is correct.

When parameter *a* increased from 0.13 to 0.18 in steps of 0.01, except for a certain degree of change in the asymptomatic undiagnosed population, the number of susceptible people, asymptomatic diagnosed population, symptomatic infected population, recovered population, and death population did not change significantly (Supplementary Figure 4), which is consistent with the sensitivity of parameter a of 0.1664 (Table 3), indicating that the parameter is a non-sensitive parameter with no significant impact on the epidemic.

When parameter *b* increased from 0.004 to 0.014 in steps of 0.002, the number of susceptible people, asymptomatic undiagnosed population, asymptomatic diagnosed population, symptomatic infected population, recovered population, and death population had no significant changes (Supplementary Figure 5), indicating that parameter *b* is a non-sensitive parameter, which is consistent with the sensitivity of parameter *b* of 1731 (Table 3).

Similarly, when parameter *c* increased from 0.005 to 0.01 in steps of 0.001, there was no significant change in the 6 groups of people (as shown in Supplementary Figure 6), indicating that parameter c is a non-sensitive parameter, which corresponds to the sensitivity of parameter *c* of 0.1757 (Table 3).

When parameter *d* increased from 0.013 to 0.018 in steps of 0.001, there were significant changes in the asymptomatic undiagnosed population, symptomatic infected population, and death population (Supplementary Figure 7), indicating that parameter d is a sensitive parameter, which is consistent with the sensitivity of parameter *d* of 0.6163 (Table 3).

When parameter *g* increased from 0.004 to 0.009 in steps of 0.001, in addition to the cured population, the number of susceptible populations, asymptomatic undiagnosed populations, asymptomatic diagnosed populations, symptomatic infected populations, and death populations all changed significantly (Supplementary Figure 8). It means that parameter *g* is a sensitive parameter that affects the epidemic, which is consistent with the sensitivity of parameter *g* of 0.521 (Table 3).

When parameter *f*_1_ increased from 0.006 to 0.011 in steps of 0.001, the number of susceptible populations, asymptomatic undiagnosed population, asymptomatic diagnosed population, symptomatic infected population, and death population all changed significantly, except for the recovered. Besides, the amplitude of change is greater than the parameters *d* and g (Supplementary Figure 9). This is because the sensitivity of parameter *f*_1_ is 0.8247, which is significantly greater than the sensitivity of parameters *d* and *g* (0.6163 and 0.521) (Table 3).

When parameter *f*_2_ increased from 0.0002 to 0.0007 in steps of.0001, except for the obvious changes in death population, there was no significant change in the susceptible population, asymptomatic undiagnosed population, asymptomatic diagnosed population, symptomatic infected population, and recovered population (Supplementary Figure 10), indicating that parameter *f*_2_ is a non-sensitive parameter, which is consistent with the sensitivity of parameter *f*_2_ (0.169) (Table 3).

In summary, we have verified the sensitivity indexes of the 10 parameters obtained by theoretical calculations through numerical simulation. The results showed that gradient change in the sensitive parameters can significantly affect the development of the epidemic, and that change in the insensitive parameters has little impact on the epidemic (only a small impact on a certain group of people or no significant impact at all). Thus, the accuracy of the parameter sensitivity analysis was proved. In addition, the higher the sensitivity of the parameter, the greater the impact of parameter changes on the epidemic.

### Influence of Parameter Changes on Attack Rate

**Figure 7:**
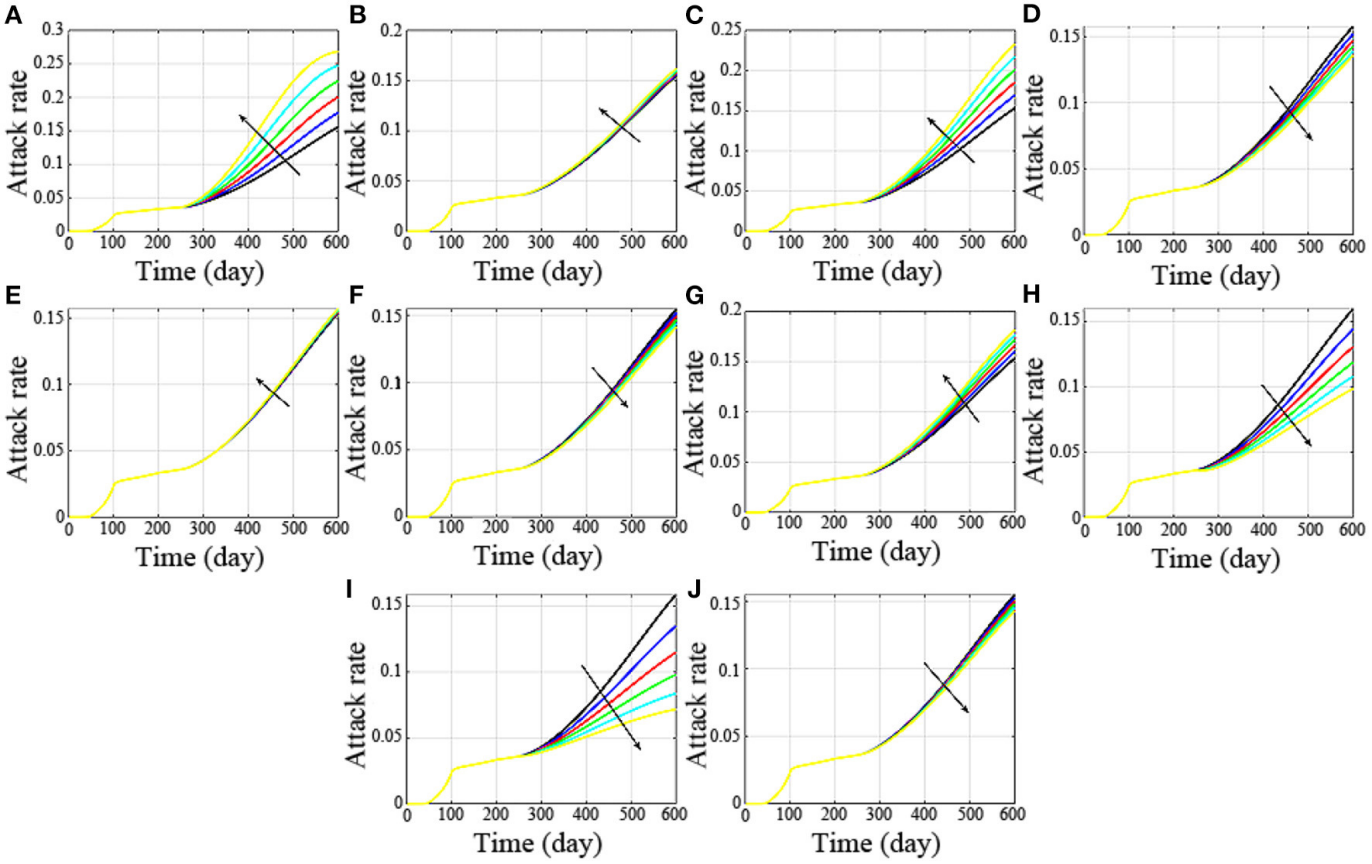
Influence of parameter changes on attack rate. **(A-J)** are the attack rates with the changes of the parameter r_1_, r_2_, r_3_, a, b, c, d, g, f_1_ and f_2_, respectively.

In addition, the influence of parameter changes on attack rate was also studied. The simulation results (Figure 7A) showed that when increase parameter r1 from 0.05 to 0.1 in steps of 0.01, the attack rate increases significantly. which was shown in Figure 7A, where the color and black arrow have the same meaning as above. Similarly, increase *r*_3_ from 0.016 to 0.021 in steps of 0.001 (Figure 7C), increase *g* from 0.004 to 0.009 in steps of 0.001 (Figure 7H), and increase *f*_1_ from 0.006 to 0.011 in steps of 0.001 (Figure 7I), the attack rates are all affected significantly. What the difference is that with the increase in *r*_1_ and *r*_3_, the attack rate also increases, and when parameters *g* and *f*_1_ increase, the attack rate decreases significantly. This is consistent with our sensitive analysis.

Besides, if *r*_2_ increases from 0.0013 to 0.0018 in steps of 0.0001, the attack rate has no significant difference (Figure 7B). Similarly, increase *a* from 0.13 to 0.14, 0.15, 0.16, 0.17 and 0.18 (Figure 7D), increase parameter *b* from 0.004 to 0.006, 0.008, 0.01, 0.012, and 0.014 (Figure 7E), increase parameter *c* from 0.005 to 0.006, 0.007, 0.008, 0.009, and 0.01 (Figure 7F), there was no significant change in their attack rates. The non-sensitive parameters are consistent with our theoretical analysis.

However, when *d* increases from 0.013 to 0.018 in steps of 0.001, the change is not particularly dramatic. This is because with the increase in *d*, the asymptomatic undiagnosed population and symptomatic infected population increased, and the asymptomatic diagnosed population decreased.

## Conclusion

Based on the transmission characteristics of COVID-19 in the population, in this study, the population was divided into 6 categories, namely, the susceptible population, asymptomatic undiagnosed population, asymptomatic diagnosed population, symptomatic infected population, recovered population, and death population, and a differential mathematical model was established to describe and predict the trend of COVID-19. Based on the real-time big data report of Baidu's epidemic situation (https://voice.baidu.com/act/newpneumonia/newpneumonia?), the number of the currently infected with COVID-19, recovered, and death from February 22, 2020 to December 1, 2020 in the United States was collected. The NLS algorithm was used to estimate the model parameters, and the correlations were calculated between the mathematical model and the collected epidemic data (the correlation between the number of the currently infected simulated by the mathematical model, and the collected infected is 98.85%, the correlation between the cured number simulated by the mathematical model and the collected cured number is 99.84%, and the correlation between the death simulated by the mathematical model and the collected death is 99.54%). Besides, we also calculated the basic reproductive number of each stage to access the transmission capacity. Subsequently, we continued to collect data (the number of the current infected, recovered and death of COVID-19 in the United States from December 2, 2020 to January 10, 2021, and verified the accuracy of the model. The results show that the prediction accuracy of the infected *I*(*t*) is 98.33%, the recovered *R*(*t*) is 98.2%, and the death *D*(*t*) is 98.96%. Therefore, this model can effectively describe and predict the evolution of the epidemic in the United States.

In order to overcome the influence of coupling effect between parameters on the results, the global sensitivity analysis method was adopted to analyze the sensitivity of the parameters, so as to obtain the sensitive parameters that affected the evolution of the epidemic, and provide effective control strategies for the prevention and control of the epidemic by adjusting the sensitive parameters.

The global sensitivity analysis results show that parameters *r*_1_ (S_Ti_ = 0.8317), *r*_3_ (S_Ti_ = 0.6427), *d* (S_Ti_ = 0.6163), *g* (S_Ti_ = 0.521), and *f*_1_(S_Ti_ = 0.8247) are sensitive parameters, and that parameters *r*_2_ (S_Ti_ = 0.2353), *a* (S_Ti_ = 0.1664), *b* (S_Ti_ = 0.1731), *c* (S_Ti_ = 0.1757), and *f*_2_ (S_Ti_ = 0.169) are non-sensitive parameters. Next, the method of parameter gradient change is adopted to verify the correctness of the parameter sensitivity results of the theoretical analysis. The results showed that change in the sensitive parameters could significantly affect change in the epidemic, and that change in the insensitive parameters had no significant impact on the epidemic. For example, the sensitivity of parameter r1 is S_Ti_ = 0.8317. When *r*_1_ increases from 0.05 to 0.1 in steps of 0.01, the number of the susceptible reduced greatly, while the asymptomatic undiagnosed, asymptomatic diagnosed, symptomatic infected, recovered, and death all increased significantly with the increase in *r*_1_. The sensitivity of parameter *r*_2_ is S_Ti_ = 0.2353. When *r*_2_ increases from 0.0013 to 0.0018 in steps of 0.0001, the number of the susceptible, asymptomatic undiagnosed, asymptomatic diagnosed, symptomatic infected, recovered, and death has no significant change.

Therefore, based on the above sensitivity analysis results, this study proposes the following control strategies:

**1. Strengthen isolation**. Because strengthening isolation can effectively reduce the infection rate of the asymptomatic undiagnosed to the susceptible (*r*_1_) and the infection rate of the symptomatic infected to the susceptible (*r*_3_). Reducing *r*_1_ and *r*_3_ can increase the number of the susceptible, and decrease the number of the asymptomatic undiagnosed, asymptomatic diagnosed, symptomatic infected, and death. In addition, if someone has to travel, wearing a mask may also be an effective isolation measure.

**2. Strengthen the monitoring and treatment of the asymptomatic diagnosed**. When the probability (*d*) that the asymptomatic diagnosed turns into the symptomatic infected increases, the number of the susceptible population will decrease, but the number the asymptomatic undiagnosed, symptomatic infected, and death will significantly increase. In addition, the recovered rate (*g*) of the asymptomatic diagnosed should be increased, because when parameter *g* increases, the number of the susceptible increases, while the number of the asymptomatic undiagnosed, asymptomatic diagnosed, symptomatic infected, and death increases significantly with the increase in *g*.

**3. Improve the recovered rate of the symptomatic infected**. When the recovered rate (*f*_1_) of the symptomatic infected increases, the number of the susceptible will increase, while the number of the asymptomatic undiagnosed, asymptomatic diagnosed, symptomatic infected and death will decrease significantly.

## Data Availability Statement

The original contributions presented in the study are included in the article/Supplementary Material, further inquiries can be directed to the corresponding author/s.

## Author Contributions

DS: conceptualization, methodology, software, funding acquisition, formal analysis, resources, investigation, writing-original draft, supervision, and writing-review editing. XL: data curation and project administration. JL: simulations, supervision and funding support. All authors contributed to the article and approved the submitted version.

## Funding

This study was supported by the National Natural Science of China under Grant Nos. 62103287 and 62003071. This study was also supported by Shenzhen Institutes of Advanced Technology Innovation Program for Excellent Young Researchers (RCBS20200714114856171).

## Conflict of Interest

The authors declare that the research was conducted in the absence of any commercial or financial relationships that could be construed as a potential conflict of interest.

## Publisher's Note

All claims expressed in this article are solely those of the authors and do not necessarily represent those of their affiliated organizations, or those of the publisher, the editors and the reviewers. Any product that may be evaluated in this article, or claim that may be made by its manufacturer, is not guaranteed or endorsed by the publisher.
